# Hydrogen in Transplantation: Potential Applications and Therapeutic Implications

**DOI:** 10.3390/biomedicines12010118

**Published:** 2024-01-06

**Authors:** Takafumi Obara, Hiromichi Naito, Tsuyoshi Nojima, Takahiro Hirayama, Takashi Hongo, Kohei Ageta, Toshiyuki Aokage, Masaki Hisamura, Tetsuya Yumoto, Atsunori Nakao

**Affiliations:** Department of Emergency, Critical Care, and Disaster Medicine, Faculty of Medicine, Dentistry, and Pharmaceutical Sciences, Okayama University, 2-5-1 Shikata-cho, Kita-ku, Okayama 700-8558, Japan; dainosinn@gmail.com (T.O.); t.nojima1002@gmail.com (T.N.); ce-hirayama@okayama-u.ac.jp (T.H.); taka.hongo123@gmail.com (T.H.); ageage1982@gmail.com (K.A.); toshiyukiaokage@gmail.com (T.A.); hisamura@msj.biglobe.ne.jp (M.H.); tyumoto@cc.okayama-u.ac.jp (T.Y.); qq-nakao@okayama-u.ac.jp (A.N.)

**Keywords:** hydrogen, organ transplantation, ischemia reperfusion

## Abstract

Hydrogen gas, renowned for its antioxidant properties, has emerged as a novel therapeutic agent with applications across various medical domains, positioning it as a potential adjunct therapy in transplantation. Beyond its antioxidative properties, hydrogen also exerts anti-inflammatory effects by modulating pro-inflammatory cytokines and signaling pathways. Furthermore, hydrogen’s capacity to activate cytoprotective pathways bolsters cellular resilience against stressors. In recent decades, significant advancements have been made in the critical medical procedure of transplantation. However, persistent challenges such as ischemia-reperfusion injury (IRI) and graft rejection continue to hinder transplant success rates. This comprehensive review explores the potential applications and therapeutic implications of hydrogen in transplantation, shedding light on its role in mitigating IRI, improving graft survival, and modulating immune responses. Through a meticulous analysis encompassing both preclinical and clinical studies, we aim to provide valuable insights into the promising utility of hydrogen as a complementary therapy in transplantation.

## 1. Introduction

The latest findings in medical research regarding hydrogen unequivocally highlight substantial prospects for harnessing hydrogen as a therapeutic intervention. Extensive observations in both clinical and experimental studies distinctly demonstrate that hydrogen holds significant promise as an innovative therapy to address unmet patient needs across various etiologies [1,2,3,4,5,6,7,8,9,10]. Renowned for its antioxidant properties, hydrogen gas has emerged as a novel therapeutic agent with potential applications in various medical domains, including transplantation. In addition to its antioxidative attributes, hydrogen also exerts anti-inflammatory effects by modulating pro-inflammatory cytokines and signaling pathways. Moreover, hydrogen’s capacity to activate cytoprotective pathways enhances cellular resilience to stress.

Due to advancements in new medications and improved surgical techniques over the past few decades, the success rate of organ transplantation has risen significantly, establishing it as the primary treatment for end-stage organ failure. However, favorable outcomes of transplant recipients continue to be hindered by persistent challenges such as ischemia-reperfusion injury (IRI) and graft rejection. Despite extensive efforts to augment the pool of deceased organ donors in response to escalating demand, the number of available cadaveric donors has remained largely stagnant. This disparity has led to thousands of preventable deaths each year due to organ scarcity.

In an effort to mitigate patient fatalities on the waiting list, strategies have been implemented to broaden the organ donor pool, including the utilization of marginal donors. The relationship between hydrogen therapy and organ transplantation is intriguing, particularly the potential of hydrogen to enhance transplantation outcomes [11].

Hydrogen plays a significant role in reducing graft rejection, improving graft survival, and modulating immune responses in transplantation, as presented in this comprehensive review. By analyzing both preclinical and clinical studies meticulously, we aim to provide valuable insight into hydrogen’s potential utility as a complementary therapy in transplantation (Table 1).

Organ grafts may experience multiple injuries during warm ischemia, cold ischemia, and reperfusion injury. These injuries all contribute to primary graft dysfunction, which is a major cause of morbidity and mortality after organ transplantation. Hydrogen therapy entails the administration of hydrogen gas or hydrogen-enriched solutions to exert therapeutic effects. In the context of organ transplantation, hydrogen, with its antioxidant, anti-inflammatory, and cytoprotective properties, has been explored as a potential strategy to address major challenges associated with organ transplantation.

This review paper explores the potential applications and therapeutic implications of hydrogen in transplantation, shedding light on its role in mitigating IRI, improving graft survival, and modulating immune responses. This review clearly demonstrates that hydrogen possesses biological effects for mitigating graft injuries and that hydrogen may have a huge potential as a safe and potent therapeutic tool for its recipients. Through an in-depth analysis of preclinical and clinical studies, we aim to provide insights into the promising use of hydrogen as an adjunct therapy in transplantation.

## 2. Challenges in Organ Transplantation

Organ transplantation failures result from a range of immunological and non-immunological factors, presenting in either chronic or acute forms. These failures are influenced by complex networks of interconnected biological events. Organ grafts may undergo injuries at different stages, including during warm ischemia, cold ischemia, reperfusion, and the acute or chronic phases. IRI arises when there is a temporary disruption of the blood supply during organ removal and subsequent reimplantation. Reperfusion, while essential for restoring blood flow and oxygen to the graft, can paradoxically trigger oxidative stress, inflammation, and tissue damage. This can compromise the function and viability of the transplanted organ. IRI contributes to primary graft dysfunction, which is a significant cause of morbidity and mortality following organ transplantation [35]. The immune responses responsible for graft rejection can imperil the success of transplants. Graft rejection is a complex process in which the recipient’s immune system detects the transplanted organ as foreign and mounts an immune response against it. This uncontrolled immune response can lead to a graft’s dysfunction and ultimate failure. Nevertheless, more research is necessary to gain a comprehensive understanding of hydrogen’s impact on immune responses in the context of transplantation. It is worth noting that long-term immunosuppressant medication may increase the risk of malignancy or infectious disease development.

Graft-versus-host disease (GVHD) primarily affects recipients of bone marrow or stem cell transplants, where the transplanted immune cells (graft) attack the recipient’s tissues and organs. This immune assault can result in inflammation and damage to various organs, including the skin, liver, gastrointestinal tract, and lungs. The immune response prompted by GVHD releases inflammatory molecules known as cytokines, which can provoke widespread inflammation which is detrimental to the function of the transplanted organ. The chronic inflammatory state induced by GVHD can lead to multi-organ dysfunction over time, impacting the overall health and function of transplanted organs. GVHD presents a substantial challenge in transplantation, prompting ongoing research to enhance prevention and treatment strategies [36,37].

Moreover, to address the current organ shortage for transplantation, the donor pool has been expanded to include marginal donors, such as elderly donors and non-heart-beating donors, and grafts subjected to prolonged cold storage [38]. However, grafts from these donors exhibit more severe graft injury and a higher propensity for failure compared to non-marginal organs. Although there are various approaches for graft protection, including pre- and post-transplant management, an optimal prophylactic treatment for graft failure remains undetermined. Consequently, long-term graft survival and function are influenced by numerous factors, including ongoing alloimmune reactions, early IRI, recipient metabolic abnormalities (elevated cholesterol or lipids), other recipient conditions (viral infections, hypertension), and adverse reactions to chronic immunosuppressive therapy. Transplant medicine is a challenging and intricate field, and specific therapeutic strategies are crucial for improving both short-term and long-term graft and patient outcomes.

## 3. Hydrogen: Chemistry and Physiology

Hydrogen (molecular formula H_2_) is the lightest molecule, an odorless, tasteless, colorless, nonmetallic, and highly combustible diatomic gas. Hydrogen is chemically stable at room temperature, which is mainly determined by the strong covalent bond between hydrogen atoms. It is known to be quite flammable and reactive with specific catalysts or in the presence of heat.

Endogenously, humans do not produce hydrogen since enzymes with hydrogenase activity are not present in the human body. However, in the large intestine, anaerobic organisms generate hydrogen by breaking down carbohydrates, primarily from the undigested polysaccharide fraction of starches and plant cells, through hydrogenase activity. Every day, the human body continuously produces several liters of hydrogen under normal physiological conditions, mainly through the fermentation of non-digestible carbohydrates by microbiota in the large intestine [39,40]. In vivo hydrogen will be released to the outside in a manner similar to circulation and respiration. Hydrogen is excreted via the lungs or through the rectum in the form of flatulence. Since hydrogen has a large diffusion capacity, the proportion of hydrogen released from the skin cannot be ignored [41]. It is noteworthy that the enterobacterial flora serves as a significant source of both hydrogen and hydrogen sulfide.

## 4. Clinical Use of Hydrogen and Toxicity

It is essential to identify safety concerns, potential side effects, and toxicity associated with the use of therapeutic gases [11,42,43]. While research is ongoing, existing evidence suggests that hydrogen therapy is well-tolerated by most individuals, with minimal to no adverse effects reported. This favorable safety profile has piqued the interest in hydrogen therapy within medical research. However, it is crucial to emphasize that hydrogen therapy, like any medical treatment, should be administered under the guidance of qualified healthcare professionals. The safety and appropriateness of hydrogen therapy may vary depending on the specific medical condition, mode of administration, and individual patient factors. As is the case with any medical intervention, a comprehensive evaluation of potential risks and benefits should be conducted, and treatment should be tailored to each patient’s specific needs. Patients should consult their healthcare providers to determine whether hydrogen therapy is a suitable option for addressing their specific health concerns and to ensure its safe and effective use. When administered under appropriate medical supervision and following established protocols, hydrogen therapy in medicine is generally considered safe [44].

There are two prominent challenges or enigmas concerning the impact of hydrogen. Firstly, the dose-dependent effect remains elusive. Whether in animal experiments or clinical observations, hydrogen is administered in small doses, yet it yields substantial effects. Secondly, both humans and animals produce a considerable quantity of hydrogen through their gut bacteria, but the following question remains: why does the increase in such a minute amount of hydrogen result in such pronounced effects? Furthermore, various issues, including the molecular mechanisms of hydrogen and the optimal methods for utilizing it in the treatment of different diseases—such as dosage, frequency, and so forth—still require further investigation [45].

## 5. Protective Mechanism of Hydrogen

### 5.1. Scavenging Free Radicals

Although various mechanisms for the cellular and tissue protection provided by hydrogen exposure have been suggested, hydrogen’s role as a scavenger of reactive oxygen species has been advocated. Ohsawa et al. reported that, in vitro, hydrogen selectively reduces peroxynitrite and hydroxyl radicals, which are very strong oxidants which react indiscriminately with nucleic acids, proteins, and lipids, resulting in lipid peroxidation, DNA fragmentation, and protein inactivation. Biochemical experiments using electron resonance spectroscopy spin traps and fluorescent probes suggest that the effects of hydrogen against hydroxyl radicals are more potent than those against peroxynitrite [46,47,48].

### 5.2. Protection of Mitochondrial Function

Hydrogen easily permeates biological membranes and diffuses into the nucleus, mitochondria, and cytosol, reaching target tissues. Treatment with hydrogen-rich saline significantly reduced the loss of mitochondrial membrane potential and preserved mitochondrial cytochrome c content [49]. In an open-label trial, Ito et al. investigated the effects of drinking hydrogen-enriched water for 12 weeks in patients with mitochondrial metabolism diseases, including progressive muscular dystrophy and mitochondrial myopathies, and observed significant improvements in lactate-to-pyruvate ratios [50]. Zhang et al. reported that hydrogen improved mitochondrial quality by upregulating heme oxygenase-1 (HO-1) expression through the nuclear factor erythroid 2-related factor 2 (Nrf2)/YY1 complex in vitro [51].

### 5.3. Anti-Inflammation

Hydrogen can modulate the production of inflammatory cytokines like interleukin (IL)-1 beta (IL-1β), IL-6, and tumor necrosis factor-alpha (TNF-α). These cytokines are key players in the inflammatory response of the human body. Hydrogen attenuates this inflammatory response by inhibiting the nuclear factor-kappa B (NF-κB) and p38 MAPK pathways [52]. Hydrogen can promote very early M1-to-M2 polarization without disturbing the functions of the M1 phenotype, suggesting that hydrogen could reduce inflammation by shifting early macrophage polarization in clinical settings [53]. Hydrogen’s anti-inflammatory impact on the inflammatory response was demonstrated to occur through the phosphatidylinositol 3 kinase/protein kinase B signaling pathway [54].

### 5.4. Induction of Antioxidant Enzyme

Another potential mechanism underlying hydrogen’s cellular protective function may be an increase in antioxidant enzymes such as superoxide dismutase, catalase, or HO-1 [55]. Hydrogen-rich saline treatment considerably increased the antioxidant enzyme levels of serum superoxide dismutase and reduced glutathione [56].

### 5.5. Induction of Surfactant-Related Genes

Tanaka et al. investigated the changes in genes after hydrogen inhalation using a gene array analysis and showed that clara cell protein 16 (CC16) was the most upregulated gene in response to preloading hydrogen in lung grafts. CC16 is one of the major proteins secreted by the respiratory epithelium and has antioxidant and anti-inflammatory properties. Preloading hydrogen via mechanical ventilation also significantly increased other surfactant-related mRNAs, including HSD11b1, SCGB3A2, and SP-A [27].

### 5.6. Protection of Vascular Endothelial Cells

Nitric oxide (NO) plays a crucial role in maintaining the delicate equilibrium of factors that regulate vascular tone, blood flow, and coagulation, all of which are vital for the health of endothelial cells [57]. Hydrogen can enhance endothelial NO synthase activity, leading to increased circulating NO levels, thus providing protection to vascular endothelial cells. The incubation in a hydrogen-rich medium significantly improves cell viability and shields human umbilical vein endothelial cells from cellular damage induced by hydrogen peroxide [29]. Moreover, hydrogen effectively suppresses the release of cell adhesion molecules like intercellular cell adhesion molecule-1 and vascular cell adhesion molecule-1, as well as proinflammatory mediators, including high-mobility group box 1 protein, IL-1β, and TNF-α. Furthermore, hydrogen demonstrates the ability to elevate levels of the anti-inflammatory cytokine IL-10 [58,59].

### 5.7. Prevention of Apoptosis

Hydrogen’s anti-apoptotic functions have been proposed to occur via the inhibition of caspase-3 activation and the induction of anti-apoptotic gene B-cell lymphoma-2 (Bcl-2) [32]. Terasaki et al. reported that the activation of the pro-apoptotic gene Bax was reduced via hydrogen treatment [60]. The anti-apoptotic effects of hydrogen gas inhalation were partially mediated by the early activation of NF-κB during hydrogen treatment and correlated with decreased levels of Bax and elevated levels of the anti-apoptotic protein Bcl-2 [61]. Zhang et al. demonstrated that the upregulation of Bcl-2, NF-κB, HO-1, and zinc finger protein A20 was seen in rats where only the donors received hydrogen [23]. Meanwhile, in another study, the intraperitoneal administration of hydrogen-rich saline to a transplant recipient immediately after reperfusion protected them against acute kidney injury after liver transplantation, partly by reducing apoptosis, which is potentially involved in the modulation of p53-mediated autophagy [21].

### 5.8. Inhibition of Infiltrating Cell Migration

Accelerated atherosclerosis caused by the immune response is a primary cause of graft loss after organ transplantation. Smooth muscle cell proliferation and migration play important roles in the progression of intimal hyperplasia. Sun et al. demonstrated that the incubation in a hydrogen-rich medium suppressed smooth muscle cell migration using an in vitro rat smooth muscle cell (A7r5) culture model [29]. Matrix metalloproteinases (MMPs) are important mediators of intimal hyperplasia, and MMP-2 and MMP-9 promote the formation of neointima. In the same study, the oral intake of hydrogen-rich water effectively inhibited MMP-2 and MMP-9 in rat vein grafts [29].

### 5.9. Inhibition of Fibrosis

Type III collagen plays a significant role in the interstitia of solid organs and in the formation of granulation tissue following ischemic tissue damage. Terasaki et al. conducted a study showing that both the inhalation of 3% hydrogen gas and the oral consumption of hydrogen-enriched water reduced oxidative stress and apoptosis, which are indicators of acute lung damage, in mice exposed to irradiation [60]. This led to a decrease in the deposition of type III collagen and the development of lung fibrosis, which is a manifestation of late-stage damage. Therefore, hydrogen’s potential to mitigate fibrosis may prove effective in addressing chronic allograft injury.

### 5.10. Immunomodulation

Hydrogen has been found to influence various aspects of the immune response. By modulating cytokine profiles and immune cell interactions, hydrogen may promote an environment conducive to immune tolerance and decrease the likelihood of graft rejection. Using a mouse model, Itoh et al. demonstrated that drinking hydrogen-rich water could attenuate an immediate allergic reaction by inhibiting the phosphorylation of FcεRI-associated Lyn and its downstream signaling molecules, which subsequently reduced the generation of hydrogen peroxide and suppressed NADPH oxidase activity [62]. Hydrogen’s immunomodulatory effects have been investigated in both adaptive and innate immune responses. Hydrogen regulates T cell differentiation, dendritic cell function, and cytokine profiles, potentially leading to immune tolerance and prolonged graft survival [22]. In another study, T cell proliferation was significantly suppressed in the in vitro presence of hydrogen and accompanied by the lowered production of interferon-ɣ and IL-2 [28].

**Figure 1:**
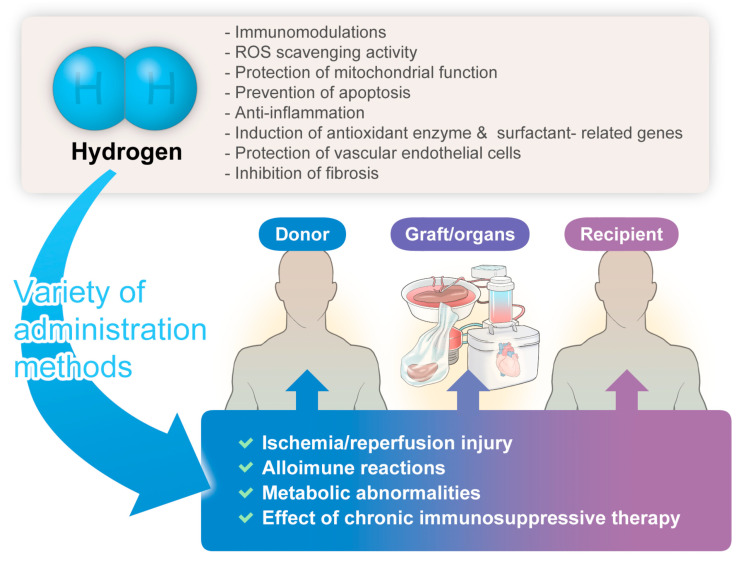
The significant benefits/potential of hydrogen gas therapy in transplantation medicine.

The hydrogen-related advantages described herein might hold significant implications for donors, grafts/organs, and recipients, manifesting anticipated organ-protective effects (Figure 1).

## 6. Hydrogen Applications in Transplantation

### 6.1. Hydrogen Gas Inhalation

Since hydrogen is a gaseous molecule, a safe concentration of hydrogen inhalation would be a straightforward delivery method for employing hydrogen as a therapeutic tool. Buchholz et al. demonstrated that hydrogen inhalation by recipients at a 2% concentration significantly dampened transplant-induced muscularis inflammation, mitigated bowel dysfunction, and prevented bowel dysmotility in rat intestinal transplant models [34]. In a lung transplantation brain death rat model, donor and recipient ventilation with 2% hydrogen hindered oxidative injuries by increasing the actions of superoxide dismutase and other antioxidants to protect lung function [63]. Kawamura et al. also indicated the efficacy of donor treatment with 2% hydrogen for three hours on lung allograft function in rats [31]. Zhang et al. demonstrated that one hour of donor treatment with 2% hydrogen inhalation significantly reduced liver injury after transplantation in a rat orthotopic liver transplant model [23].

### 6.2. Lung Inflation during Cold Preservation

Among transplantable organs, the lung is unique because it is an organ that contains air, allowing for the incorporation of hydrogen into the air within the alveoli. Lung inflation with 3% hydrogen gas during the cold ischemia phase alleviated lung graft injury, determined by inhibiting apoptosis and inflammatory responses, and improved graft function [16,64]. Similarly, Duan et al. investigated the efficacy of lung inflation during cold ischemia with 3% hydrogen + 40% oxygen + 57% nitrogen in a rat model and demonstrated that hydrogen exposure during cold ischemia improved donor lung quality by mitigating mitochondrial structural anomalies, enhancing mitochondrial function, and reducing apoptosis, inflammation, and oxidative stress, which may be achieved through activation of the Nrf2/HO-1 pathway [13].

### 6.3. Preservation Solution

The ongoing optimization of organ preservation solutions has always been an important component of donor protection for lung transplantation. Various ways of dissolving hydrogen gas in organ preservation solutions have been developed, including the use of electrolysis, a hydrogen gas cylinder, or a hydrogen-generating agent. The use of a hydrogen-rich preservation solution (more than 1.0 ppm) diminishes IRI in rat lungs during cold ischemia through anti-inflammatory and antioxidant effects [18]. Abe et al. demonstrated that 24 to 48 h of organ preservation in hydrogen-rich University of Wisconsin solution attenuated renal cold IRI in a syngeneic rat kidney transplantation model and was associated with less interstitial macrophage infiltration, tubular apoptosis, and oxidative stress in the kidney grafts, better renal graft function, and longer graft survival compared to simple cold storage [26].

Buchholz et al. indicated that luminal preservation, cold graft storage, and vascular flush in a hydrogen-bubbled preservation solution significantly preserved mucosal graft morphology and diminished graft malondialdehyde levels, showing significant reduction potential and weakened proinflammatory molecular responses within the re-perfused intestinal graft in rats [30].

Similarly, a hydrogen gas-containing organ preservation solution impeded the development of acute injuries in a donor’s kidney after cardiac death in a preclinical miniature pig model with an optimal immunosuppressive protocol based on the human clinical setting. A marginal kidney processed using a hydrogen gas-containing preservation solution was engrafted for longer than 100 days [14]. Kobayashi et al. studied a practical method of quickly dissolving hydrogen gas in organ preservation solutions using a canister containing a hydrogen-absorbing alloy [19]. After 30 min of warm ischemic injury induced by circulatory arrest, the donor kidneys were harvested and perfused for five minutes with a hydrogen-containing cold ET-Kyoto (ETK) solution in a miniature pig kidney transplantation model. Preservation in a hydrogen-containing solution for either one or four hours resulted in better renal function with more blood flow [19]. In another study, Kayawake et al. demonstrated that a hydrogen-rich preservation solution lessened IRI in a canine left lung transplantation model following 23 h of cold ischemia in an ETK solution. The graft function in this study, determined using blood gas analysis, significantly improved in the hydrogen-treated group, which was associated with lesser extents of lung edema and histopathological injury [15].

### 6.4. Hydrogen Flush after Cold Storage

Protective effects can be achieved by subjecting hydrogen to a single flush ex vivo, even without placing it in a storage solution. In a previous study, hydrogen flush after 24 h of cold ischemia significantly lowered transaminases, high-mobility group box protein 1 release, and portal venous pressure compared to vehicle-treated controls. The portal venous route maintained the sinusoidal endothelia, and the arterial route attenuated biliary damage [65].

### 6.5. Ex Vivo Perfusion

Ex vivo lung perfusion allows for the evaluation and recovery of an ex vivo donor lung by perfusion with normothermic perfusate. Haam et al. reported that lung graft ventilation after cardiac death with 2% hydrogen gas for four hours during ex vivo perfusion significantly mitigated inflammation-related lung injury and improved lung function in a porcine model [66]. Noda et al. used a rat lung ex vivo perfusion model and ventilated the lungs with air supplemented with 2% hydrogen for four hours. Hydrogen administration decreased proinflammatory changes through the upregulation of HO-1, promoted mitochondrial biogenesis, and significantly reduced lactate production. In addition, the expression of hypoxia-inducible factor-1 in the hydrogen-treated lungs was significantly attenuated. Thus, the preconditioning of lung grafts with inhaled hydrogen diminished these proinflammatory changes, promoted mitochondrial biogenesis in the lungs throughout the procedure, and resulted in better post-transplant graft function [67,68]. Ishikawa et al. reported that 90 min of reperfusion with oxygenated buffer with hydrogen at 37° on an isolated perfused rat liver apparatus significantly reduced the apoptosis, energy depletion, liver enzyme leakage, redox status, impaired microcirculation, and necrosis associated with increased bile production. The phosphorylation of cytoplasmic MKK4 and JNK were suppressed by means of hydrogen treatment [69].

### 6.6. Hydrogen Exposure Using a Hydrogen Bath

Noda et al. invented a unique cold storage device with a hydrogen-rich water bath, which enabled water saturation with hydrogen and maintained saturated hydrogen levels and a consistent temperature throughout the procedure. The grafts stored with the hydrogen-rich water bath also had a higher adenosine triphosphate content and less mitochondrial damage, which were associated with efficiently ameliorated myocardial injury [29]. The use of a hydrogen-rich water bath in which hydrogen was dissolved into a solution for liver graft tissues enabled superior morphologic and functional protection against IRI in a rat liver transplant model [20].

### 6.7. Venous/Intraperitoneal Injection

Luo et al. demonstrated that the intravenous administration of hydrogen-saturated saline to the recipient enhances the migration and proliferation capacity of bone marrow mesenchymal stem cells (BMSCs) to repair spinal cord injury by reducing the inflammatory response and oxidative stress in the injured area, suggesting that hydrogen and BMSC co-delivery is an effective method of improving BMSC transplantation in the treatment of spinal cord injury [12]. Intravenous hydrogen-rich saline administered via the tail vein at the beginning of reperfusion significantly diminished the severity of pancreatic IRI in rats, possibly by decreasing inflammation and oxidative stress [24].

### 6.8. Intraluminal Administration

The small intestine is a unique organ because it has both luminal and vascular routes through which preservation solutions can be administered. In addition to the vessel walls, the epithelial cell layers forming the mucosa and covering the inner part of the lumen are also highly susceptible to IRI and, thus, a potential therapeutic target [70]. Yamamoto et al. demonstrated that the intraluminal administration of hydrogen-rich saline regulated the loss of the transmembrane protein ZO-1 in the graft intestine and modulated IRI to the transplanted intestine in rats [17].

### 6.9. Oral Intake of a Hydrogen-Rich Solution

Oral consumption of hydrogen-rich solutions could be implemented easily into everyday clinical practice [71]. Solubilized hydrogen may be valuable since it is a safe, easily administered, and portable method of delivering hydrogen to the human body. Cardinal et al. showed that the oral intake of water containing dissolved hydrogen resulted in a sustained increase in the hydrogen levels in the serum and kidney, a better kidney allograft function over a 60-day follow-up period, and a reduction in the markers of inflammation and tissue oxidation. Noda et al. showed that drinking hydrogen-rich water prolongs the survival of cardiac allografts and decreases intimal hyperplasia in aortic allografts [66]. Furthermore, in another study, T cell proliferation was significantly restrained in the presence of hydrogen in vitro, accompanied by a lower production of interferon-ɣ and IL-2. In yet another study, hydrogen treatment was also associated with higher graft ATP levels and higher activity of the mitochondrial respiratory chain enzymes [25].

## 7. Clinical Studies

While numerous preclinical studies have showcased hydrogen’s potential in transplantation, human clinical trials remain relatively scarce. Initial clinical data indicate its safety and potential efficacy, but more extensive, controlled trials are required to firmly establish hydrogen’s therapeutic advantages. It is important to recognize that, despite the promising prospects of hydrogen in transplantation, research in this field is still in its early stages. Key considerations, such as the most effective administration method, dosage, and timing of hydrogen therapy, are pivotal aspects warranting further investigation [72,73] and must be carefully evaluated to maximize hydrogen’s therapeutic benefits while mitigating potential drawbacks. Moreover, comprehending how hydrogen interacts with existing immunosuppressive regimens is crucial for optimizing its utilization. Of note, it is important to be aware of the negative results of hydrogen therapy. Hosgood et al. showed that the administration of hydrogen gas did not increase renal function or decrease oxidative damage or inflammation during the reperfusion of kidneys with ischemia damage [74].

## 8. Future Direction

While data support the use of hydrogen in many aspects of our current transplant practices, transplant surgeons cannot assume that treatments successful in lab animals or even critically ill transplant patients will yield the same results in critically ill trauma patients. Further studies, including well-designed clinical trials and investigations exploring detailed mechanisms, are required to fully unlock hydrogen’s potential and integrate it into transplantation protocols [75,76].

The ability to administer medical gases through inhalation makes this treatment highly attractive for translation into a human clinical setting. Some gases like supplemental oxygen are already being administered in intensive care units. In most cases, medical gas treatment via inhalation can seamlessly complement existing therapeutic strategies by incorporating the gas into conventional delivery mechanism. Hydrogen’s limited reactivity with other gases at therapeutic concentrations allows it to be administered alongside other therapeutic gases, including inhaled anesthesia agents, as part of a combined gas therapy. Recent research has demonstrated that combined therapy with hydrogen and carbon monoxide (CO) enhances therapeutic efficacy over single-gas treatment in preventing cold IRI after heart transplantation, operating through anti-inflammatory and antioxidant mechanisms [33]. Meng et al. showed that lung inflation with CO or hydrogen during cold ischemia protected against IRI through anti-apoptotic, antioxidant, and anti-inflammatory mechanisms in a rat lung transplantation model, with enhanced benefits observed when CO and hydrogen were used in combination [77]. Shinbo et al. demonstrated that the inhalation of a combined gas (80 ppm of NO and 2% hydrogen) effectively mitigated myocardial infarction and improved left ventricular function in a mouse left anterior descending coronary artery ligation model, outperforming NO inhalation alone. This suggests that the inhibitory effect of NO on inflammation may be enhanced by adding hydrogen to the inhaled NO gas, thereby eliminating highly reactive byproducts like peroxynitrite [78].

## 9. Conclusions

As is clear in this review, hydrogen gas therapy may have well-defined benefits in transplant medicine. Hydrogen holds the potential to address critical challenges in organ transplantation, primarily IRI and graft rejection, through its antioxidative, anti-inflammatory, and immunomodulatory properties. However, more comprehensive research, including well-designed clinical trials, is needed to determine the safety, efficacy, and long-term impact of hydrogen therapy on transplant outcomes. At this moment, there are no available clinical studies investigating the efficacies of hydrogen in transplantation. Appropriately designed randomized controlled trials with patient-important outcomes, such as the improvement of the functioning of target organs, decreased intensive care unit and hospital stays, and decreased cost of therapy, are sorely needed to establish the role of hydrogen therapy in patients with disease and discover its advantages over preexisting standard therapies for transplant patients.

## Figures and Tables

**Table 1:** Application of hydrogen in organ transplantation models in the medical literature. Hydrogen is used in various administration routes, such as inhalation gas and as a soluble form in solutions. The effectiveness of hydrogen has been demonstrated when administered to donors, organs, or recipients. Thus, hydrogen may be used in a variety of situations and conditions in organ transplantation.

Author	Year	Target Organ	Animals	Combination	Hydrogen	Ref
Luo et al.	2023	mesenchymal stem cell	rat	syngeneic	hydrogen-rich saline (intraperitoneal)	[12]
Duan et al.	2023	lung	rat	syngeneic	3% inflation	[13]
Nishi et al.	2021	kidney	pig	allogenic	hydrogen-rich preservation solution	[14]
Kayawake et al.	2021	lung	beagles	allogenic	hydrogen-rich preservation solution	[15]
Zheng et al.	2021	lung	rat	syngeneic	3% inhalation	[16]
Yamamoto et al.	2020	intestine	rat	syngeneic	hydrogen-rich saline (intraluminal)	[17]
Saito et al.	2020	lung	rat	syngeneic	hydrogen-rich preservation solution	[18]
Kobayashi et al.	2019	kidney	pig	allogenic	hydrogen-rich preservation solution	[19]
Uto et al.	2019	liver	rat	syngeneic	hydrogen-rich preservation solution	[20]
Du et al.	2016	liver, acute kidney injury	rat	syngeneic	hydrogen-rich saline (intraperitoneal)	[21]
Yuan et al.	2015	stem cell	mouse	allogenic	hydrogen-rich saline (intraperitoneal)	[22]
Zhang et al.	2015	liver	rat	syngeneic	1–3% inhalation	[23]
Luo et al.	2015	pancreas	rat	syngeneic	hydrogen-rich saline (intravenous)	[24]
Noda et al.	2012	heart/aorta	rat	allogenic	hydrogen water oral intake	[25]
Abe et al.	2012	kidney	rat	syngeneic	hydrogen-rich preservation solution	[26]
Tanaka et al.	2012	lung	rat	syngeneic	2% inhalation	[27]
Noda et al.	2012	heart	rat	allogenic/syngeneic	hydrogen water bath	[28]
Sun et al.	2012	vein	rat	syngeneic	hydrogen water oral intake	[29]
Buchholz et al.	2011	intestine	rat	syngeneic	hydrogen-rich preservation solution	[30]
Kawamura et al.	2011	lung	rat	syngeneic	2% inhalation	[31]
Kawamura et al.	2010	lung	rat	syngeneic	2% inhalation	[32]
Cardinal et al.	2010	kidney	rat	allogenic	hydrogen water oral intake	[1]
Nakao et al.	2010	heart	rat	syngeneic	1–3% inhalation	[33]
Buchholz et al.	2008	intestine	rat	syngeneic	2% inhalation	[34]

## Data Availability

No research data were collected.
